# Comment on Kesić et al. Early Diagnostics of Vulvar Intraepithelial Neoplasia. *Cancers* 2022, *14*, 1822

**DOI:** 10.3390/cancers14205087

**Published:** 2022-10-18

**Authors:** Vincenzo De Giorgi, Elisabetta Magnaterra, Manfredi Magliulo, Flavia Silvestri, Federico Venturi, Biancamaria Zuccaro, Jacopo Colombo, Luciana Trane

**Affiliations:** 1 Section of Dermatology, Department of Health Sciences, University of Florence, 50121 Florence, Italy; 2 Cancer Research “Attilia Pofferi” Foundation, 51100 Pistoia, Italy

We have read with great interest the paper by Kesić, V. et al. entitled “Early Diagnostics of Vulvar Intraepithelial Neoplasia” [1]. This paper emphasizes the importance of early diagnosis in the management of vulvar intraepithelial neoplasia (VIN) and highlights the large problem of the differential diagnosis of such lesions. In fact, at the vulvar level, we can find a large number of lesions, both pigmented and non-pigmented, of inflammatory, neoplastic or infectious origin. The authors therefore emphasize the fact that these lesions do not clinically have specific characteristics, and it is frequently necessary to resort to histological examination for a correct diagnosis. In their accurate paper, however, the authors do not mention the use of non-invasive diagnostic methods such as dermoscopy [2,3] and confocal microscopy [4], which for years have changed the diagnostic and preoperative management of all cutaneous and mucosal lesions, with significant improvement in diagnostic accuracy even at the vulvar level. In particular, dermoscopy has become a widely used diagnostic tool because it increases the specificity and sensitivity of the examination of pigmented and non-pigmented skin and mucosal lesions as compared with the naked eye [5].

In fact, even though at present we do not yet have pathognomonic dermoscopic parameters for the diagnosis of VIN, the literature, in numerous case studies, is beginning to outline the dermoscopic characteristics that allow us to examine such lesions in their various degrees [6,7,8,9].For other lesions present at the vulvar level, even extremely malignant ones such as melanoma and squamous cell carcinoma andthose of an inflammatory type such as lichen sclerosus and or melanosis (absolutely benign pigmented lesions more frequent than all found at the vulvar level), the dermoscopic pictures are well-described in the literature and, therefore, allow a significant improvement in diagnostic accuracy, avoiding unnecessary skin biopsies [10,11,12,13].

In addition, dermoscopy can also be extremely useful in carrying out a biopsy as it allows for the selection of the point of greatest suspicion within the lesion. All this provides for a better and immediate preoperative classification of vulvar lesions, significantly reducing the number of biopsies on lesions that are then found to be absolutely benign upon histological examination [14,15].

Furthermore, in the case of topical treatment, such as imiquimod, for some forms of VIN, with dermoscopy, the effectiveness of the therapy can be evaluated over time.

For these reasons, we believe that the use of non-invasive diagnostic methods such as dermoscopy should be at least mentioned in the excellent paper by Kesić et al.
